# Storage stability of a fluidized-bed agglomerated spray-dried strawberry powder mixture

**DOI:** 10.12688/f1000research.138509.1

**Published:** 2023-09-20

**Authors:** Hader Ivan Castaño Peláez, Misael Cortés-Rodríguez, Rodrigo Ortega-Toro

**Affiliations:** 1 Facultad de Ciencias Básicas, Sociales y Humanas, Politécnico Jaime Isaza Cadavid, Medellín, Antioquia, Colombia; 2 Facultad de Ciencias Agrarias, Universidad Nacional de Colombia, Medellín, Medellín, Colombia; 3 Food engineering, Universidad de Cartagena, Cartagena, Bolívar, 130001, Colombia

**Keywords:** Fragaria ananassa Dutch, storage stability, clumping

## Abstract

**Background:** Strawberry is a fruit with a high antioxidant capacity due to its richness in phenolic compounds that suffer a rapid post-harvest deterioration. Spray drying is an alternative to reduce losses; however, these powders present problems of instantanisation, making it necessary to implement agglomeration processes. During storage, powdered food products can undergo a series of changes in their amorphous state from a product initially in a vitreous state to a gummy state, where all properties are substantially modified due to the increased mobility of water in the matrix.

**Methods:** The research objective was to evaluate the storage stability (6 months) of a fluidized bed agglomerated strawberry powder mixture at three temperatures (15, 25 and 25°C), a controlled environment at 65% relative moisture, and PET *foil* laminated film bags as packaging. Moisture, water activity, *bulk* and compacted density, Carr and Hausner indices, solubility, hygroscopicity, wettability, angle of repose, antioxidant capacities, total phenols, anthocyanins, vitamin C, color (CIE-Lab) and particle size were monitored.

**Results:** ANOVA showed statistically significant differences (p<0.05) for all dependent variables concerning storage time; storage temperature had no significant effect on S, ABTS, DPPH and Hu. The time-temperature interaction during storage had no significant effect (p>0.05) on S, ABTS, DPPH, Hu and L. The agglomerate showed moisture and aw values that confer excellent stability against deterioration reactions; it retained good fluidity, low cohesiveness, and retentions above 50% for antioxidant capacity, 76% for total phenols, 39% for anthocyanins, and 40% for vitamin C; particle size was retained during the evaluation. The color was only affected in the 35°C treatment from the fifth month onwards.

**Conclusions:** The study will serve as a tool for the determination of the shelf life of the chipboard once the critical values of the attributes selected as predictors of shelf life are defined.

## Introduction

The strawberry ( *Fragaria ananassa* Dutch.) is a plant belonging to the Rosaceae family, considered a pleasure fruit par excellence. The fruit is noted for its vitamin C (Vit.C), tannins, flavonoids, anthocyanins, catechin, quercetin and kaempferol, organic acids (citric, malic, oxalic, salicylic and ellagic) and minerals (K, P, Ca, Na and Fe, among others). These compounds in strawberries have potent antioxidant power, helping to reduce the risk of cardiovascular events, improve vascular endothelial function and decrease thrombosis ( Forbes-Hernandez et al., 2016).

Spray drying (SD) is one of the most widely used technologies in producing food powders, allowing them to increase their shelf life; however, most of these products present fluidity and instantaneousness deficiencies ( Samborska et al., 2022). These powders are mainly characterized by being very fine and with particle sizes below 100 μm, where high interparticle cohesion forces are present, especially due to Van der Waals forces, which predominate over gravitational forces by several orders of magnitude ( Rosa et al., 2020). The agglomeration process allows the bonding of solid particles improving related physical properties, such as wettability (Hu), cohesion, dispersibility, and solubility (S); in addition, it produces better rheological properties, decreasing the compaction, cohesiveness, and adhesiveness of fruit powders ( Jeong & Yoo, 2023). Fluidized bed agglomeration has the potential to achieve superior product quality in terms of oxidation stability because of the encapsulation of spoilage-sensitive compounds; retaining bioactive compounds during storage is paramount for these products' shelf life and final quality ( Haas et al., 2020).

Studies on changes in the quality of a product as a function of time are important to ensure compliance with food standards. Several factors affect the shelf life of food, such as the environmental conditions to which the food is exposed (temperature, relative humidity (RH), and presence of light); in addition, the properties of the packaging used (permeability to oxygen, water vapor and light) ( Wong & Lim, 2016). Another important result is the kinetic modeling of the change in food quality during storage, which describes the reaction rate as a function of time, and therefore allows prediction of the changes after the product starts its logistic distribution process ( Wong & Lim, 2016; Chang et al., 2018).

During storage, powdered products may experience changes in the nature of the amorphous state of matter from a vitreous to a gummy state; where the physical, chemical, sensory, microbiological, and nutritional properties are substantially modified due to the increased molecular mobility of water in the matrix ( Hernández-Sandoval et al., 2014). These changes, like the material, are caused by the adsorption of moisture from the medium and storage temperatures above the glass transition temperature (Tg) ( Rahman, 2017), resulting in progressive compaction and an irreversible state of caking ( Chang et al., 2018).

Although the integration of the SD and agglomeration processes allows high preservation of the functional properties of strawberries and improvement of the flowability and instantaneousness properties of the strawberry powder mixture, the physicochemical properties of the fruit powders during storage change due to the hygroscopicity (H) and stickiness characteristics of the particles, in response to the food-environment interaction ( Robaina et al., 2019; Samborska et al., 2022), there is evidence of many studies evaluating the storage stability of fruit powder blends: bael (Aegle marmelos) ( Sornsomboonsuk et al., 2019), papaya ( Gomes et al., 2018), coconut ( Carlos et al., 2018), soursop ( Chang et al., 2018) and blackberry. However, no reported studies evaluate the stability of fruit powder blends, especially strawberries. In this context, this study aimed to evaluate the storage stability of a strawberry powder mixture agglomerated by a fluidized bed.

## Methods

### Raw materials

Fresh strawberries ( *Fragaria ananassa* Duch, var. Monterrey) were purchased at the wholesale market in Medellín and refrigerated at 4 °C until use was used. The degree of ripeness of the strawberries corresponded to a scale of 5-6 according to the Colombian Technical Standard NTC 4103. Additionally, gum Arabic (GA) (Tic Pretested gum Arabic FT Powder, Tic Gums, USA) and maltodextrin (MD) with dextrose equivalent 19-20 (Ingredion) were used as drying additives.

### Preparation of the feed suspension for the spray dryer

Strawberries without stalks and sepals were washed and disinfected with Citrosan® (0.25% v/v). They were then processed in an IKA colloid mill, model MK 2000/5, coupled to a water-cooling system (≅ 5 °C) (3660 rpm, minimal clearance of the grinding disc, flow rate: 240 mL/minute), until a homogenized strawberry pulp (HSP) with total solids (TS) of 8.9% was obtained; this pulp was stored frozen (-18 °C) until use. An industrial batch of 100 kg of feed suspension SD (SSD) with a total TS content equivalent to 19.6% was prepared under the following procedure: 1) thawing of HSP, 2) weighing of ingredients: HSP with a contribution of 8.9% of the ST of the SSD, GA (0.22% w/w) and MD (11.5% w/w), and 3) slow addition of the GA and MD under homogenization in an Ultra Turrax, IKA - UTL 50 (10000 rpm and time = 5 minutes). The development of the strawberry base colloidal suspension formulation without including MD and its processing has been reported by Castaño-Peláez et al., 2022a.

### Spray drying process

The SD process was carried out in an industrial tower (Lemar, China, with a water evaporation capacity of 200 kg/h), model LPG320, co-current flow, and operating under sub-atmospheric conditions. The process conditions were air inlet temperature (154 °C), air outlet temperature (89 °C), and atomizer disc speed (15000 rpm). The formulation of the colloidal suspension of feed to the SD (MD included) and the drying process conditions was established from the investigation at the pilot level, reported by Castaño-Peláez et al., 2022b; where the air inlet and outlet temperatures were kept at the pilot level; while the speed of the industrial spray disc (rpm) was recalculated, keeping the same tangential speed used in the pilot equipment (Vibrasec spray dryer, model PASALAB 1.5).

### Agglomeration process

A Lemar (China) fluidized bed agglomerator, model FL5, with a 3000 g charge and operating at a fluidizing air temperature of 70 °C, binder solution atomization pressure of 1 bar, binder solution flow rate of 10-15 mL/min at 25 °C and with a vitamin C (Vit.C) concentration of 3.33 g/L and a blower frequency of 30-34 Hz was used. SiO _2_ (Pirosil®) at 0.5 w/w was used to improve the fluidization of the filler in the agglomeration process. The basis for the operating conditions in the FL5 agglomerator equipment was established from the research work reported by Castaño-Peláez et al., 2022c.

### Storage

The storage study of the strawberry agglomerated powdered mix (StPM) was carried out using a completely randomized factorial design, considering two independent variables: temperatures (T) (15, 25 and 35 °C) and time (t): 0, 30, 60, 90, 120, 150 and 180 days, and the dependent variables: moisture (Xw), water activity (a _w_), solubility (S), wettability (Hu), hygroscopicity (Hy), bulk density (ρ _b_), compacted density (ρ _c_), Carr's index (CI), Hausner's index (HI), angle of repose (AR), particle size in terms of equivalent surface diameter (D _[3;2]_), antioxidant activity (ABTS and DPPH methods), total phenols (TF), Vit. C, total anthocyanins (TA) and color in CIE-L*a*b* space. Samples were packed in laminated PET film bags, aluminum foil with O _2_ permeability <1 mL/(m ^2^*24h*atm), water vapor permeability < 1 g/(m ^2^*24h*atm), ALICO® brand, and stored in climate chambers conditioned with a controlled relative humidity (RH) of 65%. The dependent variables were evaluated from three replicates per storage condition.

### Characterization of StPM properties

Xw was determined according to the official AOAC method ( 2003); a _w_ was determined using a dew point hygrometer at 25 °C (Aqualab 3TE series, Decagon). The ρ _b_ was determined according to the methodology described by Pereira et al., (2020), modified by weighing 5 g of sample and recording the volume occupied in a test tube. The ρ _c_ was determined according to the methodology described by Haas et al. (2020) modified, by weighing 5 g of powder in a falcon tube, then centrifuging for 5 min at 8000 rpm, and finally, the compacted volume was recorded. The CI and HI parameters were determined according to equations 1 and 2, respectively, whose flowability and cohesiveness classification ( Table 1) was described by ( Jinapong et al., 2008).

$$\mathrm{CI}=100\left(\frac{\rho_{C}-\rho_{b}}{\rho_{C}}\right)$$ (Equation 1)

$$\mathrm{HI}=\left(\frac{\rho_{C}}{\rho_{b}}\right)$$ (Equation 2)

**Table 1.** Classification of fluidity and cohesiveness of powders according to CI and HI.

Fluency	CI (%)	Cohesiveness	HI
Very good	<15	Low	< 1.2
Good	15-20	Intermediate	1.2-1.4
Acceptable	20-35	High	>1.4
Poor	35-45	---	---
Very poor	>45	---	---

AR was determined according to the methodology described by ( Barbosa-Cánovas & Juliano, 2005). S was determined according to the method described by Marulanda et al. (2018), 1g of product was weighed and dispersed in 50 mL of water. The mixture was centrifuged at 3000rpm/5min at 25°C, and 25mL of the supernatant was taken and dried in an oven at 105°C for 5 hours. The S (%) was calculated as the difference between the initial and final weight of the dried. Hu was determined as the time required for 1 g of powder to disappear from the surface of a 100 mL volume of water at 20 °C ( Marulanda et al., 2018). Hy was determined according to the gravimetric method for sorption isotherms by controlling the ambient RH inside an airtight bottle at 68% (supersaturated KI solution). Particle size was determined on the Mastersizer 3000 particle analyzer (Malver Instrument), Aero S module, and reported as equivalent surface area diameter (D _[3;2]_). TF content was determined by the colorimetric method using phosphomolybdic-phosphotungstic acid reagents ( Singleton & Rossi, 1965). The extract obtained from the powdered agglomerate was mixed with a methanolic solution of the 2,2-Diphenyl-1-picrylhydrazyl (DPPH°) radical and reacted for 30 minutes to evaluate the antioxidant activity. The reduction in DPPH° absorbance was measured at 515 nm, and the percentage inhibition of the radical was calculated. Trolox was the positive control ( Brand-Williams et al., 1995). Likewise, the antioxidant activity was determined by the ABTS method, where performed radical cation of 2,2′-azinobis-(3-ethylbenzothiazoline-6-sulfonic acid) (ABTS•+) was generated through the oxidation of ABTS with potassium persulfate. The ABTS•+ was subsequently reduced in the presence of the extract obtained from the powdered agglomerate. The extent of radical discoloration was quantified as a reduction in absorbance of the radical cation at 734 nm, and this reduction was calculated as the percentage inhibition of ABTS•+. Trolox was employed as the reference standard ( Re et al., 1999).

Vit.C was determined by HPLC (Shimatzu Prominence 20A), column: Luna® 5 um C18(2) 100A - 250*4.6 mm, mobile phase: KH _2_PO _4_ 0.02M - pH: 3.06, flow: 1 mL/min, pressure: 1172 psi, retention times: 4.317-4.456 min, injection volume: 5 μL and wavelength: 244 nm. An analytical standard L-Ascorbic acid (Sigma Aldrich 47863, Lot LRAC1812) was used. The extraction process was performed by weighing 0.25 g of StPM in a graduated test tube and adding a 0.02M KH _2_PO _4_ buffer solution adjusted to pH 3.0 with 85% orthophosphoric acid to make up 20 mL. The mixture was vortexed for 2 min, centrifuged at 5000 rpm, 4 °C and 15 min, and the supernatant was passed through a 0.45 μm acetate-cellulose membrane filter and made up to 25 mL. The results were expressed in mg/100g bs.

The quantification of TA in the form of Cyanidin-3-glucoside (C _3_G) was performed by HPLC (Shimadzu), DAD-Uv-vis detector (200-700 nm), C18 column and mobile phase acetonitrile/water/formic acid (80:18:2), in a gradient mode at a constant flow rate (0.5 mL/min). The extract for TA quantification was obtained by weighing 0.25 g of sample in 50 mL falcon tubes, adding 25 mL of the solvent (1% HCl in methanol), placing in an ultrasonic bath for 15 min, centrifuged at 8000 rpm (Universal 320) for 15 min at 25 °C, filtered on 0. 45 mm in a 50 mL volumetric balloon, washed three times with 10 mL of the solvent, repeating the operations of the ultrasonic bath, centrifugation, filtration, and finishing with gauging with the HCl/methanol mixture. The sample was filtered by syringe and 0.25 uL filter in an amber vial. The results were processed with Chromeleon 7.2. software (Dionex, Thermo Scientific, United States), using chromatograms at 517 nm, expressed as mg C _3_G/100g bs.

### Data analysis

Data were analyzed from ANOVA, using the LSD (Least Significant Difference) method for multiple comparisons, with a confidence level of 95%, and using STATGRAPHICS XVI software (Statpoint Inc, USA).

## Results and Discussion

**Figure 1.**
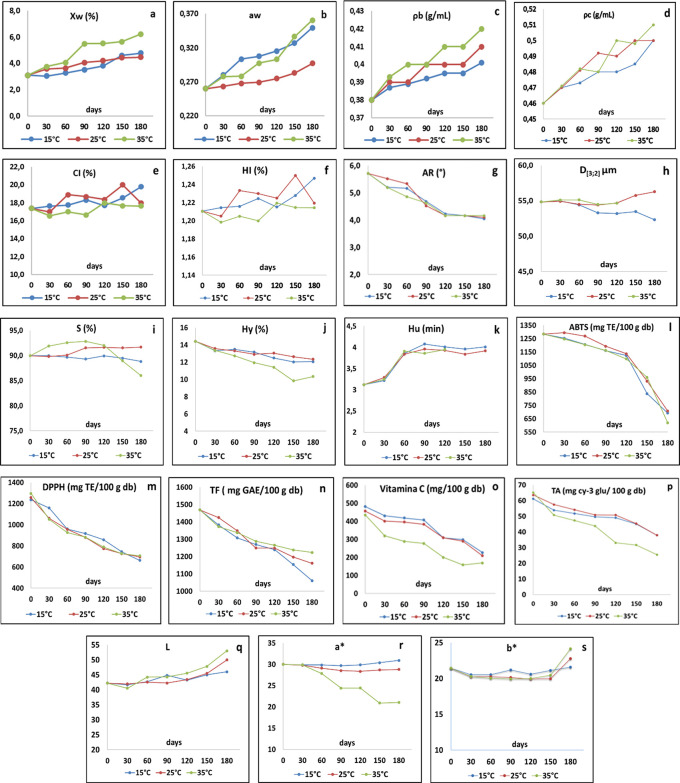
Evolution over time (days) of StPM quality attributes during storage.

Figure 1 and Table 2 present the behaviors during storage of the StPM quality attributes, and the values of the p-value statistic derived from the ANOVA, respectively. The ANOVA showed significant differences (p<0.05) of the variables Xw, a _w_, Hy, ρ _b_, ρ _c_, CI, HI, AR, D _[3;2]_, TF, DPPH, Vit. C, TA, L*, a*, and b* concerning T; while all the dependent variables presented significant differences (p<0.05) concerning t. On the other hand, the variables Xw, a _w_, Hy, S, Hu. ρ _b_, ρ _c_, CI, HI, AR, D _[3;2]_, Vit. C, L*, a*, and b* presented significant differences (p<0.05) concerning the T-t interaction.

**Table 2.** ANOVA results for StPM storage (p-values).

Source	Xw	a _w_	ρ _b_	ρ _c_	CI	HI	S	Hy	Hu
**T**	0.0000	0.0000	0.0000	0.0000	0.0000	0.0000	0.0713 *	0.0000	0.0598 ^*^
**t**	0.0000	0.0000	0.0000	0.0000	0.0000	0.0000	0.0000	0.0000	0.0048
**T-t**	0.0000	0.0000	0.0000	0.0000	0.0000	0.0000	0.0248	0.0000	0.0051

**Table 2.** ANOVA results for StPM storage (p-values).

Source	ABTS	DPPH	TF	AR	D _[3;2]_	L *	a *	b *	Vit. C	TA
T	0.8485 *	0.0214	0.0383	0.0000	0.0781	0.0257	0.0000	0.0132	0.0000	0.0000
t	0.0000	0.0000	0.0000	0.0000	0.8345 *	0.0000	0.0000	0.0000	0.0000	0.0000
T-t	0.7479 *	0.1813 *	0.1205 *	0.0000	0.7634 *	0.0262	0.0000	0.0003	0.0000	0.4990 *

* Not significant. Continued.

### Moisture and water activity

The mean values of Xw and a _w_ during storage at 180 days reached maximum values of 4.79±0.06, 4.48±0.20, 6.23±0.11%, and 0.350±0.009, 0.287±0.018, 0.361±0.006 at the temperatures of 15, 25 and 35 °C, respectively ( Figure 1a-b); values that confer excellent stability against physicochemical and microbiological deterioration reactions ( Varastegani et al., 2019). Xw presented an increasing behavior over time, being, in general, higher with increasing T. The behavior of aw was corresponding to that of Xw; however, these behaviors were not consistent concerning T, which could be attributed to the microstructural heterogeneity of the agglomerates, where the binder solution does not homogeneously impregnate the active points of the StPM surfaces, some agglomerates concerning others, which affects the levels of water adsorption during storage. In general, Xw and aw are critical variables involved by T, t, and T-t, whose behavior is considered as the result of two phenomena mainly: 1) The environment-packaging-StPM interaction at the conditions of the study generated a driving force in favor of Xw adsorption on the StPM, due to the chemical potential difference between the interior of the chamber (a _w_: 0.650) and the surface of the StPM (a _w_ < 0.361) ( Marulanda et al., 2018; Chang et al., 2018), and 2) Higher storage temperature generates higher partial pressure of water at the StPM surface, which contributes to lower Xw content.

Storage of StPMs at 25 °C was the most favorable temperature for the sixth month of control (< Xw and a _w_), with values of 4.45% and 0.297, respectively. This Xw value is like the monolayer moisture in dry products and is very suitable for food preservation ( Varastegani et al., 2019). It is highlighted that the results in the present study were lower than those reported for soursop powders obtained by SD ( Chang et al., 2018) and similar to those reported by Varastegani et al., (2019) on black cumin powder obtained by SD and by Wong & Lim, (2016) on papaya powder obtained by SD.

### Flow Properties

The values of ρ _b_ and ρ _c_ varied between 0.3753±0.003-0.420±0.000 and 0.463±0.001-0.509±0.015 g/mL, respectively. Although these variables presented significant differences concerning T, t and T-t interaction, in general, their variations were small (≅ 5%) ( Figure 1c-d), behavior that has also been reported during storage for black cumin powders obtained by SD ( Varastegani et al., 2019). On the other hand, it is highlighted that the values of the present study were similar to those reported by Oliveira et al., (2013) during storage of strawberry powder obtained by SD. Lower variations of the densities were observed at low T (15 and 25 °C), which is explained due to the small changes of Xw during storage, whereas, at 35 °C, this variation becomes larger and consistent with the increase of Xw content (higher molecular mobility of water in the agglomerate structure), which could generate changes of the non-thermodynamic state (rubbery amorphous) and higher plasticity of the material ( Liu et al., 2017).

According to the variations of the CI values (%) found (16.606±0.005-20.001±0.888), the flowability of the material during storage was little affected, retaining good flow characteristics. The cohesiveness of the agglomerate during storage through monitoring at HI allowed identifying that the material retains a low - intermediate cohesiveness (1.198±0.136-1.250±0.018), an important aspect that positively impacts the instantaneity of the material. The CI and HI variables showed an increasing trend but fluctuated concerning t. When applying Fischer's test, storage at 35 °C showed a higher significance concerning storage at 15 and 25 °C, showing at 35 °C a better fluidity and cohesiveness of the agglomerate and, at the same time, an increase in the densities ( Figure 1e-f).

The monitoring of the AR behavior during storage identified the preservation of the agglomerate fluidity by decreasing with increasing t; however, this variation was low (5.7° → 4.1°), corresponding to a change rate of 28.1%. These low RA values are favored by the presence of SiO _2_ in the StPM, which has fluidizing and anti-compacting properties, reducing the likelihood of particles sticking together by reducing the cohesive forces, thus reducing potential caking ( Chang et al., 2018). Additionally, it was observed from month 4 and in all T that AR values tend to be asymptotic ( Figure 1g); however, this behavior is in contrast with what was reported by Wong & Lim, (2016) in papaya powder obtained SD. A similar situation was observed in D _[3;2]_, whose maximum variation was between 56.3 and 52.3 μm; however, this dependent variable did not show a well-defined trend concerning the independent variables considered ( Figure 1h).

It is highlighted that from month 5 the StPM stored at 35 °C presented a caking of the material in the form of the lump (D _[3;2]_ >>>) or the possible start of crystallization of the fructose and glucose present in the StPM ( Wong & Lim, 2016), with the respective loss of agglomerate structure, which could favor the increase in particle size due to: 1) change of the non-thermodynamic state from vitreous amorphous to rubbery amorphous ( Saavedra-Leos et al., 2018); aspect related to the high Xw contents (> 5.5%) discussed above at this T and t, and 2) the interaction Xw- pectic content of the food matrix that favors inter-particle forces ( Einhorn-stoll, 2018), which is why it was only plotted up to day 120.

### Instantaneity properties

The instantaneity of food powders is a property highly valued by consumers and industry when used as a raw material in producing other food products. Instantaneity is made explicit by assessing S, Hy, and Hu properties ( Dhanalakshmi et al., 2011). The S of agglomerates is directly related to properties affecting the microstructure of the material, where the higher the degree of preservation of the vitreous state, the better the conditions for wetting, sinking, and dispersion; a collapsed structure in a rubbery state restricts the diffusional processes of water in the aggregate ( Bhandari et al., 2013).

In general, the S of StPM presented an excellent water solubility during storage (86.0-92.8%), being very stable during 6 months at 15 and 25 °C ( Figure 1i) (correlated with the stable behavior observed in properties such as ρ, AR and Hy) ( Hogekamp & Schubert, 2003). Similar S values have been reported by Varastegani et al., (2019) on black cumin powder (92.6 and 91.9%), stored at 4 °C for 12 months and, using Gum Arabic and Maltodextrin as drying agents.

On the other hand, the S of the StPM stored at 35 °C after month four shows an accelerated decrease associated with the increase in Xw and the aforementioned caking. According to the results found for the densities at this T and t, their values were higher, an indicator of the compaction experienced by the material, a negative aspect for a good interaction of the material with water in the solubilization process ( Chang et al., 2018). These authors have identified reductions of 35% of S in the storage of SD soursop powders using aluminum foil PET laminated film packaging at 35 °C. On the other hand, in the storage stability study of papaya powder obtained by SD at 38±2 °C and 90% relative humidity, using aluminum PET *foil* laminated film packaging, S decreased in week 7 from 97.1 → 85.1% ( Wong & Lim, 2016), which represents a more significant reduction compared to the findings of the present study.

The packaging material plays an important role as a barrier to water vapor, O _2,_ and CO _2_, which is why the choice of material is a fundamental decision to guarantee the stability of the product during storage. In general terms, a decrease in the Hy of the StPM during storage was observed, with the statistical analysis describing two homogeneous groups: 1) 15 and 25 °C and 2) 35 °C, where the rate of change was (14.4 → 12.1%), (14.4 → 12.3%) and (14.4 → 10.3%), respectively ( Figure 1j). The decrease in StPM Hy is a favorable aspect for the stability of the material and its shelf life; this phenomenon can be explained by the formation of a surface layer on the agglomerate structure in response to the interaction of the adsorbed water with the pectin, gum Arabic and maltodextrin of the StPM, which generates a barrier for the adsorption and subsequent diffusion of water into the matrix. The behavior of these results was contrary to those reported by Wong & Lim (2016) on papaya powder obtained by SD. It was stored for seven weeks, 38.2 °C and RH 90%, in aluminum *foil* PET (8% increase) and PET (13% increase) laminated film packaging.

Hu showed an increasing trend with t, independent of T; however, at 35 °C from five months onwards, it could not be determined due to caking problems of the StPM, discussed above ( Figure 1k). The behavior of Hu during storage was not favorable due to its increase (3.12±0.01 → 4.01±0.05 min), which contributed to a decrease in the reconstitution capacity of the material in water by 28.5%. This situation is consistent with the observed increases in ρ _b_ and ρ _c_ caused by moisture adsorption in the packaging, which reduces the material's porosity, affecting its capillarity and increasing the wetting time ( Cuq et al., 2013). No evaluations of Hu stability studies of SD powders or agglomerated powders during storage are reported in the literature to contrast the findings.

Generally, these values are favorable when at least minimal agitation is performed in the reconstitution system. On the other hand, these Hu values of the StPM compared to those of the strawberry powder mixture dried by SD and used in the binder loading (Hu: 11-19 min) show a significant reduction and preservation during storage, which translates into an improvement in the instantaneousness of the product.

### Antioxidant capacity and total phenols

In general, the behavior of ABTS, DPPH, and, TF decreased with t, the latter being the most critical variable during storage ( Figure 1l, m-n); furthermore, ANOVA showed that T exerts a greater affectation mainly on DPPH and TF.

For ABTS, it is observed that during the first 4 months, there is a low rate of change due to the good level of protection of the MD and GA on these components; subsequently, a higher rate of degradation was observed, mainly at T of 35 °C, which corresponds to the caking discussed above due to the possible change in the state of matter (vitreous → gummy), which increases the molecular mobility of water and degradation phenomena. Oliveira et al., (2013) reported preserving the antioxidant capacity of strawberry powder obtained by SD during the first 90 days of storage at 25 °C, using laminated PET film bags and aluminum *foil.*

Regarding DPPH, similar behavior is observed for 15, 25 and 35 °C, showing a linear trend with slopes of 3.155, 3.013 and 3.039 (mg TE/day) (average degradation rate = 3.069 mg TE/day); however, it is considered that the level reached at 180 days represents an important nutritional contribution. The literature does not report comparative values on DPPH for agglomerated products; no effect of T on DPPH stability during storage of Bael powder obtained by SD has been reported ( Sornsomboonsuk et al., 2019).

The results at the end of storage showed a similar level of preservation for ABTS and DPPH concerning the study T (15, 25, and 35 °C), being of the order of (53.9, 55.1 and 48.2%) and (53.6, 55.3 and 54.4%), and with correlation coefficients of 0.70, 0.62, and 0.63, respectively. The Xw further favors this preservation and a _w_ conditions reached by the products, which slow down the deterioration reactions ( Hedegaard & Skibsted, 2013). On the other hand, the presence of SiO _2_ contributes to an increase in the Tg of the StPM (45 °C), which guarantees adequate matrix stability, mainly at 15 and 25 °C, where (Tg - T _storage_) ≥ 20 °C ( Chang et al., 2018). The ABTS and DPPH preservation levels in the present study are higher than those Varastegani et al., (2019) reported in black cumin powders obtained by SD, stored for 12 months at 4 °C and RH of 40 and 60% (33 and 29%, respectively).

The stability of TF during storage is an important aspect of quality assurance of food products, which modern consumers highly appreciate. Strawberries are a rich source of phenolic compounds, specifically flavonoids, the most representative being catechin, quercetin, and anthocyanins, which are responsible for metal chelation and antioxidant capacity. Generally, high retention percentages were presented at 15, 25, and 35 °C during the 180 days of storage: 76.8, 79.9, and 83.2%, respectively. The degradation was a consequence of the statistical effect of T and t, which favors oxidation processes ( Varastegani et al., 2019); furthermore, the higher retention of TF with increasing T could be attributed to a possible higher extraction of these compounds or the hydrolysis of condensed phenolic compounds when subjecting the StPM to these conditions ( Tonon et al., 2010).

### Vitamin C

The results obtained for vitamin C during storage at 15, 25, and 35 °C showed a good regression fit for zero-order kinetics (R ^2^: 0.941, 0.910, and 0. 940 respectively), showing 2 homogeneous groups, one at 15 and 25 °C, and the other at 35 °C, with lower vitamin C levels at all control times at higher T and t; i.e. with degradation kinetics for 15-25 °C of 1.28 mg Vit. C/100 g bs x day and for 35 °C of 1.44 mg Vit. C/100 g bs x day ( Figure 1o). This situation revalidates an increased sensitivity to temperature and oxidation reactions of vitamin C at high t and T, as reported by Lucas-Aguirre et al., (2020).

The retention levels of vitamin C in the StPM at t = 0 corresponded to an average of 110.8 mg Vit. C/25g serving, corresponding to 123.1% of the recommended dose according to the World Health Organization (WHO), whereas, at month 6 for the homogeneous groups 15-25 and 35 °C, the levels reached 51.9 and 39.6 mg Vit. C/25 g serving (58 and 44% of the recommended dose), respectively. It is noted that 25 g of StPM dissolved in water to complete 250 g corresponds to a beverage with a soluble % solids content of ≅ 10%.

Degradation of the vitamin is due to the highly reactive structure of enediol; the main degradation route is oxidation to dehydroascorbic acid and subsequent oxidation of the latter compound to 2,3-dicetogulonic acid, which has no biological activity. Depending on the system conditions, 2,3-dicetogulonic acid is cycled through Strecker degradation. It produces carbon dioxide and furfural; the latter polymerizes to form melanoidins, compounds responsible for non-enzymatic browning. This mechanism is considerably complicated in the presence of reducing sugars and amino acids that favor various degradation pathways.

In the storage of acerola and Camucamu powders obtained by SD at 30 °C, 75% RH, and 30 days, vitamin C reductions of 78 and 39.5%, respectively, were observed ( dos Santos et al., 2020). Another study reported increased sensitivity of vitamin C in microcapsules in Taro starch at 50 °C with increasing RH (13, 22, 46, 57, and 72%) ( Hoyos-Leyva et al., 2018).

### Anthocyanins

Anthocyanins are the substances of the group of phenolic compounds responsible for the characteristic coloring of strawberries; cy-3-glucoside (cy-3-glu), pg-3-glucoside, and Pg-3-rutinoside stand out. Color changes in food products during storage depend on independent variables such as T, t, RH, packaging atmosphere, and packaging, among others, which contribute to a greater or lesser extent in the deterioration reactions of the matrix ( Ramachandra & Rao, 2013).

The behavior of TA at 15, 25 and 35 °C was similar to that of vitamin C, showing a decrease with increasing storage t and T, presenting a good regression fit for zero-order kinetics (R ^2^: 0.9922, 0.949, and 0.9953 for 15, 25 and 35 °C, respectively); additionally, they presented 2 homogeneous groups: 15-25 °C and 35 °C, and degradation rates of 0.116 and 0.204 mg TA/100g bs x day respectively. Similar behavior of TA in bayberry powder obtained by SD has been reported by Mahdavi et al., (2016); however, some research has reported first-order kinetics for TA, for example, Mahdavi et al., (2016) on blackberry powder obtained by SD and stored for 5 months in environments at 25 and 35 °C and an RH of 32.8%.

The retention levels of TA in the StPM at t = 0 corresponded to a mean of 63.3±2.0 mg cy-3-Glu/100g bs (15.3 mg cy-3-Glu/25 g serving), whereas, at month 6 for the homogeneous groups 15-25 °C and 35 °C, the levels reached 38.0 and 25.5 mg cy-3-Glu/100g bs (9.1 and 6.0 mg cy-3-Glu/25 g serving). Thermal degradation of anthocyanins is associated with the formation of Maillard reaction products (furfural and hydroxymethyl furfural), which condense with the anthocyanins, forming brown-colored compounds ( Delia et al., 2019). This situation is considered to have occurred in the StPM at 35 °C mainly and more intensely from day 150, where the color change from a soft pink (t=0) to camel (t > 150 days) became noticeable. This situation parallels other changes observed under these conditions: higher Xw content and development of a caked structure. This same behavior was reported in corozo ( *Acrocomia aculeata*) microcapsules obtained by SD and stored at T > 50 °C (shade red → brown) for 40 days ( Osorio et al., 2010); however, other investigations did not find significant changes in TA in SD strawberry powders during storage at 25 °C, 90 days, using aluminum *foil* PET film laminated packaging and placed in a desiccator with silica gel ( Oliveira et al., 2013).

### Color

The stability of food color during storage depends on several factors: temperature, time, relative humidity, light, humidity and composition of the product, and atmosphere (O _2_, N _2_, modified atmosphere), among others. Color is the first quality parameter the consumer observes; therefore, its assessment is essential.

The changes of the color parameters observed for 15 °C and 25 °C during storage (0 →180 days) were low: ∆L* (0.81 and 4.76), ∆a* (-0.06 and 1.81), and ∆b* (0.14 and 1.32), respectively. This situation at these conditions defines these parameters as non-critical for StPM, given that the rate of change is less than 5, not even perceptible by the human eye, as has been reported by several authors: in grape ( De Souza et al., 2015) and avocado extracts ( Marulanda et al., 2018).

**Figure 2.**
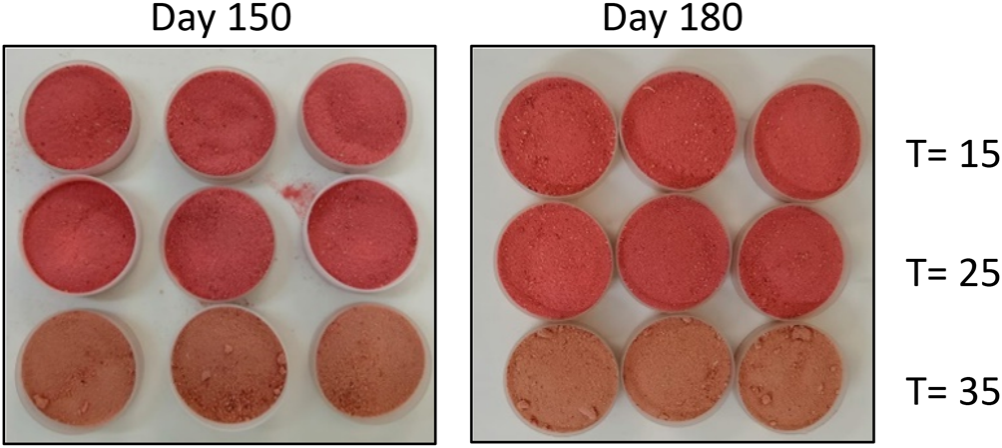
Color of agglomerates in storage at 150 and 180 days at temperatures (T) of 15, 25 and 35 °C.

Concerning storage at 35 °C, ∆L* (7.8), ∆a* (-10.0) and ∆b* (2.7) were reached, reflecting the following phenomenologist: 1) a higher clarity of the samples with the storage t, with a higher rate of change after 150 days; this situation is explained by the decrease of the reddish pigments due to their degradation, which causes the contents of the other components of the StPM: MD, SiO _2_ and GA (white powders) bring out their greater clarity ( Zanoni et al., 2020) 2) degradation of the pigments responsible for the reddish chromaticity of the StPM (anthocyanins, flavonols, among others) ( Weber et al., 2017), and possible condensation reactions of these with the products of the Maillard reactions, which finally produces a camel shade ( Figure 2). 3) a slight increase in yellow chromaticity; however, this change is insignificant (∆b* < 5).

Increasing storage temperature is a critical factor in stability; notable color changes have been reported in fruit powders obtained by SD and stored at 35 °C: corozo ( Osorio et al., 2010), blackberry ( Weber et al., 2017) and purple cabbage ( Zanoni et al., 2020).

## Conclusion

The StPM presented good physicochemical stability against deterioration reactions due to the low values of Xw and aw reached, which also confers microbiological stability (a _w_ < 0.361 and Xw < 6.2%). The stability achieved in the properties: ρ _b_, ρ _c_, S, Hy, Hu, AR, CI, HI and D _[3;2]_ confers to the food product homogeneous particle size, with good flowability and instantaneity, and low cohesiveness, mainly at 15 and 25°C. At 35°C, caking was observed from month five onwards, mainly caused by the Xw levels and the possible change from amorphous vitreous to a rubbery state. In general, the color corresponded to the above properties, with similar behavior at 15-25°C during the 180 days of storage, whereas, at 35°C, a change in the shade of the StPM was observed at 150 days: pink → camel. Good retention of bioactive compounds was achieved during storage: antioxidant capacities > 50%, TF > 76%, TA > 39%, and Vit. C > 40% (58 and 44% DR according to OMS for 15-25°C and 35°C, for 180 days). Finally, the research defined a storage t of the StPM of 180 days at 15 and 25°C, and 120 days at 35°C, using an aluminum *foil* PET laminated film bag as packaging. The study will serve as a tool for the determination of the shelf life of the chipboard once the critical values of the attributes selected as predictors of shelf life are defined.

## Data Availability

Figshare: Evolution over time (days) of StPM quality attributes during storage. DOI: https://doi.org/10.6084/m9.figshare.23915901.v1 Data are available under the terms of the Creative Commons Attribution 4.0 International license (CC-BY 4.0).
