# Supplementary material for: Storage stability of a fluidized-bed agglomerated spray-dried strawberry powder mixture
Source: F1000Res. 2023 Sep 20;12:1174. [Version 1] doi: 10.12688/f1000research.138509.1 (PMC11200056; doi:10.12688/f1000research.138509.1)
Supplement: Peer review history

# Reviewer report: Reviewer response for version 1

**DOI:** 10.5256/f1000research.151711.r232901

**Published:** 2024-06-25

**Authors:** Ramachandra C T

**Affiliations:** 1 University of Agricultural Sciences Bangalore, Bangalore, India

The manuscript is well written with a focus on spray fluid-bed dried strawberry powder mixture.

 1. What is shelf life of the chipboard mentioned in the conclusion part of the abstract?

 2. Arrange key words in alphabetical order

 3. What is the predicted shelf life over observed shelf life of the product?

Is the work clearly and accurately presented and does it cite the current literature?

Yes

If applicable, is the statistical analysis and its interpretation appropriate?

Yes

Are all the source data underlying the results available to ensure full reproducibility?

Yes

Is the study design appropriate and is the work technically sound?

Yes

Are the conclusions drawn adequately supported by the results?

Yes

Are sufficient details of methods and analysis provided to allow replication by others?

Yes

Reviewer Expertise:

Food Process Engineering; shelf life studies, post harvest engineering

I confirm that I have read this submission and believe that I have an appropriate level of expertise to confirm that it is of an acceptable scientific standard.

# Reviewer report: Reviewer response for version 1

**DOI:** 10.5256/f1000research.151711.r267506

**Published:** 2024-05-16

**Authors:** Martha Rascón Díaz

**Affiliations:** 1 Alimentos, Universidad Veracruzana,, Xalapa,, Mexico

This study evaluates the storage stability of a mixture of strawberry powder agglomerated in a fluidized bed at three temperatures with a controlled environment at 65% relative humidity and laminated film bags with PET foil as packaging. The study is interesting but has some problems:

 1. The authors must correct the errors of the finger. For example, in the methods section of the abstract, the temperature of 25°C is repeated.

 2. The authors must correct the errors of citing. For example, the authors assure that most spray-dried (SD) products present deficiencies in fluidity and instantaneity, citing the study carried out by Samborska et al., 2022. Still, this information does not exist in said study.

 Furthermore, the authors state that the powders obtained by spray drying are mainly characterized by being very fine and with particle sizes less than 100 μm, where high interparticle cohesion forces are present, mainly due to Van der Waals forces and cite a study carried out by Rosa et al., 2020; but again, this information is not found in that study.

 3. It is necessary to improve the writing of the conclusions. What the authors mean by "(58 and 44% DR according to OMS for 15-25°C and 35°C, for 180 days)"

Is the work clearly and accurately presented and does it cite the current literature?

Partly

If applicable, is the statistical analysis and its interpretation appropriate?

Yes

Are all the source data underlying the results available to ensure full reproducibility?

Yes

Is the study design appropriate and is the work technically sound?

Yes

Are the conclusions drawn adequately supported by the results?

Partly

Are sufficient details of methods and analysis provided to allow replication by others?

Yes

Reviewer Expertise:

Stability and bioaccessibility of systems carrying bioactive compounds (microencapsulation and nanoencapsulation).

I confirm that I have read this submission and believe that I have an appropriate level of expertise to confirm that it is of an acceptable scientific standard, however I have significant reservations, as outlined above.

# Reviewer report: Reviewer response for version 1

**DOI:** 10.5256/f1000research.151711.r207975

**Published:** 2023-10-27

**Authors:** Marianela Hazel Álvarez Hernández

**Affiliations:** 1 Independent researcher, Orléans, France

The manuscript presents a correct approach and is quite interesting. Indeed, strawberries are a highly perishable product. In fresh form, its shelf life is about one week under refrigeration.

 The authors mention that spray drying is one of the most common methods for producing food powders. However, the resulting products have certain deficiencies. Therefore, they propose the fluidized bed agglomeration process for developing a strawberry powder mixture.

 The authors concluded that the fluidized-bed agglomerated strawberry powder mixture retained a good physicochemical quality for up to six months at both 15 and 25 °C due to its low humidity and water activity. Nevertheless, at 35 °C the mixture developed a cake state. 

 The methodology is well-detailed. The statistical analysis is appropriate, but standard deviation is missing from graphs and decimal points must be replaced where applicable. It is not explicitly stated what the control was. Do you have any data available for strawberry powder that has been obtained exclusively through spray drying? The chart title "Vitamina C" should also be translated. In the ANOVA table, it is preferable to indicate the significance for P ≤ not signiﬁcant, 0.01, and 0.001. 

 In general, the letter "w" in the designation a _w_ for water activity should be maintained throughout the text as a subscript. It is also important to preserve the homogeneity regarding numbers and units. In this case, I recommend placing a space between the numerical value and its corresponding unit symbol.

 I consider it relevant to mention the following publications: https://doi.org/10.1016/j.powtec.2019.03.010, 10.1016/j.heliyon.2023.e19577 and 10.3233/JBR-220036.

 Please, condense and clarify sentences for better readability. I dare to suggest the following wording in the abstract: "The research aimed to analyze the storage stability of a mixture of fluidized bed agglomerated strawberry powder for six months at three different temperatures (15, 25, and 35 °C). The environment was controlled at 65% relative humidity, and PET foil laminated film bags were used for packaging. Moisture, water activity, bulk density, ..., and particle size were monitored to assess the storage stability of the powder mixture.".

 In the abstract section, the conclusion should be improved. The final conclusion of the manuscript should avoid abbreviations to facilitate understanding.

Is the work clearly and accurately presented and does it cite the current literature?

Yes

If applicable, is the statistical analysis and its interpretation appropriate?

Yes

Are all the source data underlying the results available to ensure full reproducibility?

Yes

Is the study design appropriate and is the work technically sound?

Yes

Are the conclusions drawn adequately supported by the results?

Yes

Are sufficient details of methods and analysis provided to allow replication by others?

Yes

Reviewer Expertise:

Agri-food science and technologies, postharvest conservation techniques.

I confirm that I have read this submission and believe that I have an appropriate level of expertise to confirm that it is of an acceptable scientific standard, however I have significant reservations, as outlined above.

## References

[1] : Stability of microencapsulated strawberry flavour by spray drying, freeze drying and fluid bed. *Powder Technology* .2019;347: 10.1016/j.powtec.2019.03.010 179-185 10.1016/j.powtec.2019.03.010

[2] : Optimization of fluidized bed agglomeration process for developing a blackberry powder mixture. *Heliyon* .2023;9(9) : 10.1016/j.heliyon.2023.e19577 e19577 10.1016/j.heliyon.2023.e19577 37809402 PMC10558842

[3] : Fluidised bed agglomeration of strawberry powder mix obtained for spray drying. *Journal of Berry Research* .2022;12(4) : 10.3233/JBR-220036 495-512 10.3233/JBR-220036
